# Commentary: Cultural adaptation and content validation of the Watson Caritas patient instrument for a Latin American Spanish context

**DOI:** 10.1177/17449871251333623

**Published:** 2025-08-01

**Authors:** Albert Westergren

**Affiliations:** Professor and Healthcare Strategist, Faculty of Health Sciences, Kristianstad University, Sweden;; Skåne Region, Office for Psychiatry, Habilitation and Technical Aids, Sweden

Commentary on: Cultural adaptation and content validation of the Watson Caritas patient instrument for a Latin American Spanish context (Mayut Delgado-Galeano et al., 2025).

The study titled ‘Cultural adaptation and content validation of the Watson Caritas patient instrument for a Latin American Spanish context’ presents a rigorous validation of the Watson Caritas Patient Score (WCPS) for Spanish-speaking hospitalised patients (Delgado-Galeano et al., 2025). The five-item WCPS is designed to measure the transpersonal Caritas relationship, a fundamental concept in Jean Watson’s Caring Science Theory, which emphasises compassionate, patient-centred nursing care (Brewer and Watson, 2015; Watson, 2018).

The research involved a methodological validation study, including cultural adaptation, content validity assessment, and psychometric evaluation through exploratory factor analysis (EFA) and the Rasch model. The results indicate strong reliability (Cronbach’s α of 0.84–0.86) and a single-factor structure with an explanatory power of 65%. Rasch analysis further not only confirmed the instrument’s validity but also highlighted the need for refinements, such as adjusting the response categories (1 = never; 2 = rarely; 3 = sometimes; 4 = often; 5 = always) and adding more questions to enhance measurement precision.

## Connection to nursing theory, practice and research

This study is particularly relevant in the context of modern nursing practice, which increasingly prioritises patient-centred and humanised care. Watson’s Caring Science Theory underscores the importance of intentional and compassionate nurse–patient relationships (Watson, 2018). The Rasch model, used in the study, applies a probabilistic approach in modern test theory and offers several advantages over classical test theory (CTT), which primarily relies on correlations (Andrich, 2004; Andrich and Marais, 2019). The validation of the WCPS in Spanish is a significant step towards integrating theoretical nursing frameworks into practice across diverse linguistic and cultural settings.

From a psychometric perspective, the use of the Rasch model in this study is noteworthy. As a robust approach for instrument validation, it ensures that the WCPS measures the intended construct – transpersonal Caritas relationships – consistently across populations. The study’s findings align with previous research validating the WCPS in other languages, reinforcing its applicability in diverse healthcare settings.

The study found that collapsing response categories improved the instrument’s performance, suggesting that the original five-point Likert scale might introduce unnecessary variability.

## Practical implications: who can benefit from this research?

The validated WCPS instrument has far-reaching implications for clinical practice, nursing education and healthcare policy:

*Clinical practice* – Nurses can use the WCPS to assess and enhance the quality of caring relationships in hospital settings. The tool provides a structured means of evaluating patient experiences, fostering improvements in compassionate care delivery (Feo et al., 2020).
*Nursing education* – The study reinforces the importance of integrating Watson’s Caring Science Theory into nursing curricula. Training future nurses to utilise validated instruments such as the WCPS could strengthen their ability to provide patient-centred care (Fawcett and DeSanto-Madeya, 2012).
*Healthcare policy and quality assurance* – Hospital administrators and policymakers can leverage the WCPS as a metric for assessing patient satisfaction and care quality. The study’s findings highlight the necessity of culturally adapting assessment tools to ensure their relevance in specific healthcare contexts (Wild et al., 2005).

## Areas for further research

Whilst the study successfully validates the Spanish WCPS, it also identifies areas for further refinement. Future research should focus on:

- Expanding the number of questions to improve measurement coverage.
- Evaluating the instrument in broader clinical settings, including primary care and community health.
- Conducting longitudinal studies to assess the WCPS’s ability to track changes in nursing practice over time.

## Conclusion

The study contributes significantly to the empirical application of Watson’s Caring Science Theory by providing a validated Spanish version of the WCPS. This instrument holds great potential for enhancing nursing practice, ensuring that compassionate, patient-centred care remains a fundamental priority in healthcare. Moving forward, continued refinement and application of the WCPS will be essential for fostering a culture of humanised care in nursing across diverse populations.

## References

[bibr1-17449871251333623] Andrich D (2004) Controversy and the Rasch model: A characteristic of incompatible paradigms? Medical Care 42(Suppl.): I7–16. 10.1097/01.mlr.0000103528.48582.7c 14707751

[bibr2-17449871251333623] Andrich D Marais I (2019) A Course in Rasch Measurement Theory: Measuring in the Educational, Social and Health Sciences. Singapore: Springer.

[bibr3-17449871251333623] Brewer BB Watson J (2015) Evaluation of authentic human caring professional practices. Journal of Nursing Administration 45: 622–627. 26502069 10.1097/NNA.0000000000000275

[bibr4-17449871251333623] Fawcett J DeSanto-Madeya S (2012) Contemporary Nursing Knowledge: Analysis and Evaluation of Nursing Models and Theories. Philadelphia, PA: F.A. Davis Company.

[bibr5-17449871251333623] Feo R Conroy T Wiechula R , et al (2020) Instruments measuring behavioural aspects of the nurse-patient relationship: A scoping review. Journal of Clinical Nursing 29: 1808–1821. 31162861 10.1111/jocn.14947

[bibr6-17449871251333623] Mayut Delgado-Galeano M Villamizar-Carvajal B Ibáñez-Alfonso L-E , et al. (2025) Cultural adaptation and content validation of the Watson Caritas patient instrument for a Latin American Spanish context. Journal of Research in Nursing 30: 568–585. DOI: 10.1177/17449871251333620 PMC12317968 40762013

[bibr7-17449871251333623] Watson J (2018) Unitary Caring Science Philosophy and Praxis of Nursing. Louisville, CO: University Press of Colorado.

[bibr8-17449871251333623] Wild D Grove A Martin M , et al (2005) Principles of good practice for the translation and cultural adaptation process for patient-reported outcomes (PRO) Measures: Report of the ISPOR Task Force for Translation and Cultural Adaptation. Value Health 8: 94–104. 15804318 10.1111/j.1524-4733.2005.04054.x

